# Nurse Managers’ Emotional Intelligence and Effective Leadership: A Review of Current Evidence

**DOI:** 10.2174/1874434601812010225

**Published:** 2018-10-14

**Authors:** Mohammad Al-Motlaq

**Affiliations:** Faculty of Nursing, Hashemite University, Zarqa, Jordan

## 1 INTRODUCTION

The review that was conducted by Dr. Prezerakos [1] has contributed to the body of knowledge on Emotional Intelligence (EI). It was systematically conducted with the aim to highlight the importance of EI in achieving effective nursing leadership. Although several reviews were conducted on EI in the nursing context [2-4], this was a unique study targeting nursing management and leadership. The review provided a critical analysis of the presented literature and included both qualitative and quantitative studies. While a limitation, the data presented in the review are still valuable given the lack of randomized trials on EI and nursing leadership. Prezerakos’s article is not a systematic review, though it has summarized and provided basic evidence about the significance and influence of EI on effective leadership in nursing. The outcomes ascertaining the association of EI with effective leadership do not only exist in the health sector but also other organizations and sectors [5]. Therefore, the results of Prezerakos’s article could be of benefit to a wide range of disciplines involving leadership research. In this sense, the discussion of the article was circling around the importance of emotional skills of leaders in achieving a healthy work environment [6]; see also studies in Dr. Prezerakos' review.

If we agree that EI is a viable concept which differs from the concept of personality, and if we agree on the validity and reliability of the different measures and operational definitions of the concept, we must agree that it can be an indicator of success and efficacy (see list of instruments used to measure leadership in [7], and the Situational Awareness and Emotional Intelligence survey designed by Wanda Curlee and Marie Sterling [8]). Leaders in health education and health services have introduced the concepts of EI into their work environments influencing the culture of the organization [9]. Prezerakos' review showed leaders the collective benefits of improving their leadership abilities by including EI in their plans for nurses. Understanding and improving nurses' behaviors are crucial for best patient care.

### 2 EI as a Skill

EI is a concept where the skill lends itself to complement all other skills whether in health care or any other discipline. It is of importance to note that the previous decades witnessed an increase in the conduction of research investigating EI and its associated factors within different disciplines including nursing. Research outcomes showed that EI influences nurses' work and relationships with patients. This applies to all nursing levels starting from students, professional nurses in practice, finalizing with nursing administrators, faculty members and leaders. It is well established that patient outcomes can be improved if health care professionals show empathy and resilience towards their patients. As such, emotionally intelligent managers empower their teams hence improve patient satisfaction. Actually, the expansion of research outcomes in all directions make enough bases for shifting to such evidence-based- emotional intelligence practice [2]. As a cumulative skill, EI of leaders combined their previous experiences as students and practicing nurses into their new role as managers or leaders. Leaders' efficacy cannot be judged by looking at their past undertakings but their current skills are for sure affected by their past experiences. While each leader has his/her unique personality that shapes their vision, hence their leadership style, the key to any successful leader will be gaining intercultural competency which, for sure, requires high EI [10]. In this ever-changing world, technical skills alone are no longer sufficient. Every individual including leaders needs to possess some other non-technical skills such as intercultural awareness and EI.

### 2.1 How to Improve EI of Fellow Nurses and Leaders

Although nursing is a challenging profession and dealing with everyday professional hardships can add to this challenge, nurses continue to show the ability to cope and change the challenge into an opportunity for improvement; the exact meaning of resilience which means the ability to cope with the challenging and stressful situations not by avoiding or preventing them but by strengthening the capacity to deal with them. This also applies to nursing leaders. The review by Prezerakos's highlighted the reasons why EI is important for achieving effective nursing leadership. However, in the absence of a unified theoretical and operational definition of EI, some researchers still argue the ability of a person to change their “emotional” abilities [11] while others [12] confirmed possibility to improve an individual's EI through educational intervention and empirical training.

Many factors could play a role in shaping one's values, attitudes and behaviors such as age, gender, culture, ethnicity, spirituality and life experiences [13]. However, the cumulative effect of a lifetime of insight, intuition, context, experience and wisdom helps shape one's nontechnical skills including EI, cultural sensitivity and situation awareness. Collectively, these skills can be improved with some sort of intervention. Emotional Intelligence programs [12], for example, enhanced nurses' reactions in different situations and improved patient-staff communication. Leaders of different health care services need to recognize the important role such personal traits have on the healthcare process as it is considered one part of the organization's efforts to develop intercultural competency. In addition, several courses provide student nurses with social-emotional learning where cultural competency and emotional intelligence are considered significant skills. Resilience is also a necessary trait for nursing students to possess or develop in order to succeed in study and practice [3]. Moreover, clinical communication ability of nurses to listen, respond effectively and convey information to the patients and their families is a crucial factor in patient care [14]. On the other hand, leaders and nurses could benefit from experiences with culturally diverse patients and students. Leaders wishing to plan continuing education offerings and courses should consider Cultural Competence and EI education through meaningful experiences. Well, known examples to this concept are the character of the TV series Doc Martin. Doc Martin either has emotions and empathy but usually doesn't show them in an expected way or have an emotional or attitude problem. This could also apply to the story of Cecil in Professor Goleman's book “EI”.
